# Supplementary material for: Comparison of new and emerging SARS-CoV-2 variant transmissibility through active contact testing. A comparative cross-sectional household seroprevalence study
Source: PLoS One. 2023 Apr 24;18(4):e0284372. doi: 10.1371/journal.pone.0284372 (PMC10124829; doi:10.1371/journal.pone.0284372)

# Appendix

## Supplementary information

STATA code for primary analysis

mfp, df(agenew diffpcrsero dayshh imd_rank close_contact:4): xtlogit nc_pos vocnew sexnew agenew diffpcrsero dayshh imd_rank close_contact, re or nolog

mfp: xtlogit infected vocnew sexnew agenew diffpcrsero dayshh imd_rank close_contact, re or nolog

Supplementary Table 1. Baseline characteristics in 454 participant contacts with and 385 without serum samples

|  | | Participants without serum samples | Participants with serum samples |
| --- | --- | --- | --- |
| Number of index - n  Alpha  non-VOC  total | | 162  38  200 | 188  50  238 |
| Number of contacts - n | | 385 | 454 |
| **Index characteristics^1^** | |  |  |
| index case female - n (%) | | 220 (57.1%) | 255 (56.2%) |
| Index case age – median (IQR) | | 51 (40- 64) | 57 (47-70) |
| ethnicity | white (%) | 27 (54) | 96 (51) |
|  | Asian (%) | 2 (4) | 14 (8) |
|  | Black (%) | 7 (14) | 34 (18) |
|  | Middle Eastern (%) | 5 (10) | 15 (6) |
|  | SE Asian (%) | 7 (14) | 13 (7) |
|  | other | 2 (4) | 8 (4.3) |
| Hospital site | Hospital 1 | 49 (48) | 173 (49) |
|  | Hospital 2 | 53 (52) | 179 (51) |
| household size^1^ – median (IQR) | | 3 (2-4) | 3 (2-4) |
| index case respiratory symptoms – n (%) | | 35 (70) | 146 (78) |
| symptom duration in days – median (IQR) | | 7 (3-10) | 7 (4-12) |
| index case hospitalisation – n (%) | | 29 (58) | 124 (66) |
| index case ICU admission – n (%) | | 5 (17) | 35 (29) |
| index case mortality – n(%) | | 1 (2) | 2 (1) |
| IMD decile (IQR) | | 3 (2-6) | 3 (2-5) |
| Time since index PCR diagnosis - median (IQR) | | 139(111-164) | 130  (101-158) |
| **Contact characteristics^2^** | |  |  |
| contact female – n (%) | | 172 (45) | 204 (58) |
| contact age – median (IQR) | | 25 (12-45) | 42 (24-60) |
| days of exposure to index – median (IQR) | | 5 (2-10) | 7 (3-14) |
| proximity to index – n (%) | no close contact | 86(22) | 49(14) |
|  | assisted in personal care | 167 (43) | 130 (37) |
|  | shared bedroom | 37 (10) | 59 (17) |
|  | shared bathroom | 97 (25) | 114 (32) |
| other covid exposure (not index) – n (%) | | 26 (26) | 107 (30) |
| previous COVID-19 diagnosis – n (%) | | 48 (47) | 235 (67) |
| Contact symptoms - n (%) | | 40 (39) | 183 (52) |
| Long COVID - n (%) | | 4 (8) | 8 (4) |
| vaccination status at time of serum sampling | unvaccinated | 260(68) | 169 (48) |
|  | single vaccination | 68(18) | 102 (29) |
|  | double vaccination | 57(15) | 81 (23) |

^1^ The index characteristic figures in the table refer to the number of household contacts for whom the corresponding index case had each given characteristic

^2^ The contact characteristics figures in the table refer to the number of household contacts with each given characteristic

Supplementary Figure 1 Supplementary Figure 2


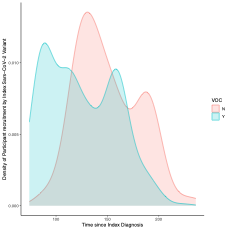

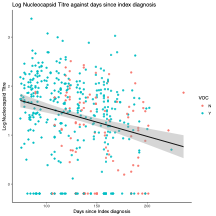


Supplementary Figure 1. Density plot showing participant recruitment from time of index PCR diagnosis by SARS-CoV-2 variant of their index case.

Supplementary Figure 2. Log normalised anti-NC SARS-CoV-2 antibody titre in household contact against time since index PCR SARS-CoV-2 diagnosis

Supplementary Figure 3. Calculating the secondary outcome with infection defined as either anti-NC IgG seropositivity in any subject or anti-spike IgG seropositivity in an unvaccinated subject with S positive unvaccinated results


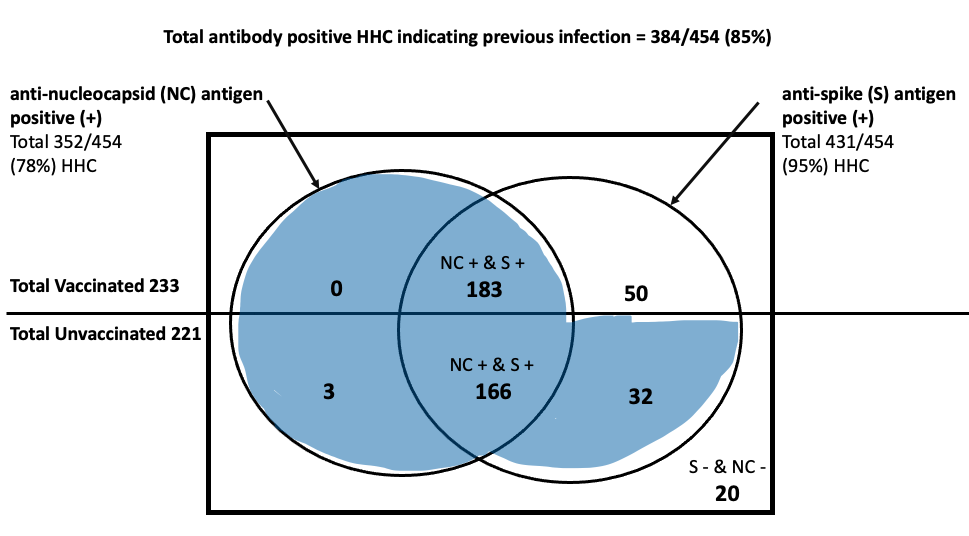

Supplement: S1 Appendix — (DOCX) [file pone.0284372.s001.docx]
