# Supplementary material for: Comparison of new and emerging SARS-CoV-2 variant transmissibility through active contact testing. A comparative cross-sectional household seroprevalence study
Source: PLoS One. 2023 Apr 24;18(4):e0284372. doi: 10.1371/journal.pone.0284372 (PMC10124829; doi:10.1371/journal.pone.0284372)

Contents

1. Summary of IMD_rank at household-level, by VOC exposure 2
2. Summary of potential confounders by (i) VOC status and (ii) nc_pos 3
3. Summary of VOC and nc_pos - unadjusted 11
4. Summary of VOC and nc_pos – adjusted (with continuous covariates grouped – diffpcrsero [3 levels]; dayshh [4 levels]; imd [4 levels]) 12
5. Summary of VOC and nc_pos – adjusted (with continuous covariates + age assessed using fractional polynomals); no adjustment for close_contact 13
6. Summary of VOC and nc_pos – adjusted (with continuous covariates + age assessed using fractional polynomals); plus adjustment for close_contact (assumed as factors) 14
7. Summary of VOC and nc_pos – adjusted (with continuous covariates + age assessed using fractional polynomals); adjustment for close_contact (assuming quantitative) 15
8. Summary of VOC and nc_pos 16
9. Summary of potential confounders with infection 17
10. Summary of VOC and infection - unadjusted 23
11. Summary of VOC and infection – adjusted (with continuous covariates + age assessed using fractional polynomals); plsu adjustment for close contact (as a factor) 24
12. Summary of VOC and infection – adjusted (with continuous covariates + age assessed using fractional polynomals); plus adjustment for close contact (as a quantitative variable) 25
13. Summary of VOC and infection – adjusted (with continuous covariates + age assessed using fractional polynomals); no adjustment for close contact 26
14. Summary of VOC and infection 28

1. Summary of IMD_rank at household-level, by VOC exposure

```
.         table vocnew if n1==1, c(median imd_rank p25 imd_rank p75 imd_rank n imd_rank)
```

vocnew	med(imd_rank)	p25(imd_rank)	p75(imd_rank)	N(imd_rank)
Non-VOC	8899	4748	21971	55
VOC	8860	5146	15029	221

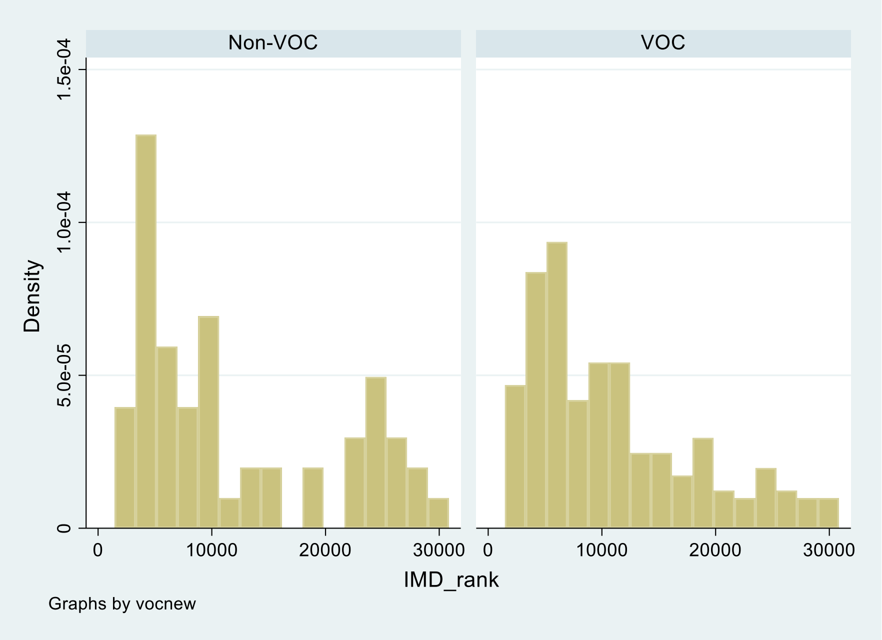

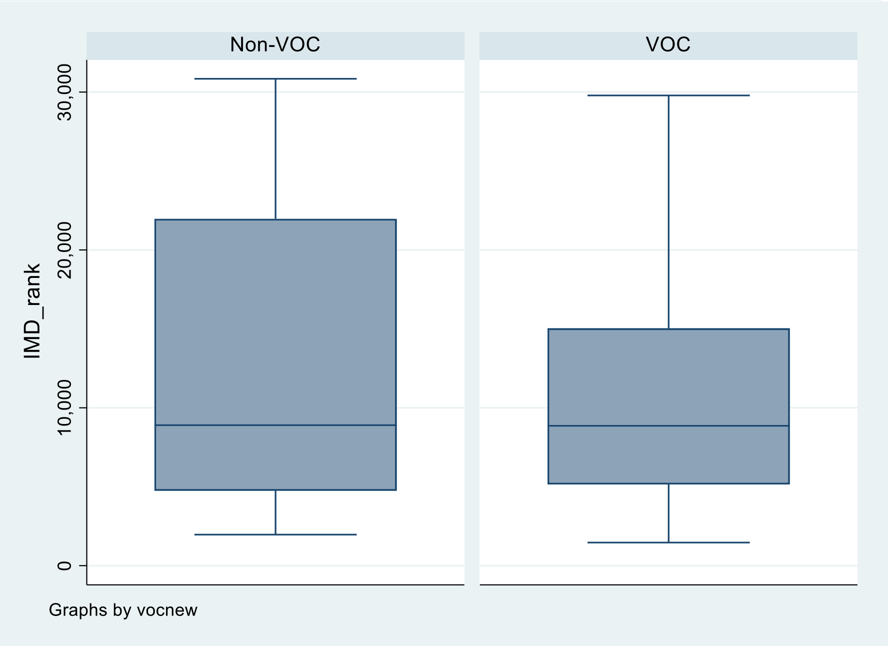

2. Summary of potential confounders by (i) VOC status and (ii) nc_pos

```
-----------------------------------------------------------------------------------------------
-----------------------------------------------------------------------------------------------
----------------------
      name:  <unnamed>
       log:  C:\Users\eidekfie\research\covid\UK\data\202206\datasummary-20220607.log
  log type:  text
 opened on:   7 Jun 2022, 11:45:25

. xtset hh_id
       panel variable:  hh_id (unbalanced)

. * data summaries
. * note cont vars: agenew, dayshh, diffpcrsero
. foreach var in sexnew imdnew isolatenew close_contact diffpcrsero_cat3 dayshh_cat {
  2.
.         tab `var' vocnew, col nokey
  3.         tab `var' nc_pos, row nokey
  4.         xtlogit nc_pos i.`var', re or nolog
  5.         quadchk, nooutput
  6. }

           |        vocnew
    sexnew |   Non-VOC        VOC |     Total
-----------+----------------------+----------
      male |        45        148 |       193
           |     44.12      42.05 |     42.51
-----------+----------------------+----------
    female |        57        204 |       261
           |     55.88      57.95 |     57.49
-----------+----------------------+----------
     Total |       102        352 |       454
           |    100.00     100.00 |    100.00

           |        NC_pos
    sexnew |       Neg        Pos |     Total
-----------+----------------------+----------
      male |        43        150 |       193
           |     22.28      77.72 |    100.00
-----------+----------------------+----------
    female |        59        202 |       261
           |     22.61      77.39 |    100.00
-----------+----------------------+----------
     Total |       102        352 |       454
           |     22.47      77.53 |    100.00

Random-effects logistic regression              Number of obs     =        454
Group variable: hh_id                           Number of groups  =        276

Random effects u_i ~ Gaussian                   Obs per group:
                                                              min =          1
                                                              avg =        1.6
                                                              max =          7

Integration method: mvaghermite                 Integration pts.  =         12

                                                Wald chi2(1)      =       0.07
Log likelihood  = -235.48501                    Prob > chi2       =     0.7859

------------------------------------------------------------------------------
      nc_pos | Odds Ratio   Std. Err.      z    P>|z|     [95% Conf. Interval]
-------------+----------------------------------------------------------------
      sexnew |
     female  |    .922787   .2729933    -0.27   0.786     .5167562    1.647848
       _cons |   5.847051   1.841601     5.61   0.000     3.153846     10.8401
-------------+----------------------------------------------------------------
    /lnsig2u |   .8131777   .5141728                     -.1945824    1.820938
-------------+----------------------------------------------------------------
     sigma_u |   1.501687   .3860632                      .9072918    2.485488
         rho |    .406689   .1240663                      .2001384    .6525099
------------------------------------------------------------------------------
Note: Estimates are transformed only in the first equation.
Note: _cons estimates baseline odds (conditional on zero random effects).
LR test of rho=0: chibar2(01) = 12.76                  Prob >= chibar2 = 0.000
```

```
Refitting model intpoints() =  8
Refitting model intpoints() = 16
```

Quadrature check

	Fitted quadrature 12 points	Comparison quadrature 8 points	Comparison quadrature 16 points	
Log likelihood	-235.48501	-235.49215	-235.48515	
		-.00713367	-.0001428	Difference
		.00003029	6.064e-07	Relative difference
nc_pos: 1.sexnew	-.0803568	-.08055042	-.08034954	
		-.00019362	7.261e-06	Difference
		.00240952	-.00009036	Relative difference
nc_pos: _cons	1.7659374	1.7674262	1.7657873	
		.0014888	-.00015006	Difference
		.00084307	-.00008498	Relative difference
/: lnsig2u	.81317773	.81656234	.81289198	
		.00338462	-.00028575	Difference
		.00416221	-.0003514	Relative difference

imdnew	vocnew Non-VOC	VOC	Total
1st	29	43	72
	28.43	12.22	15.86
2nd	3	34	37
	2.94	9.66	8.15
3rd	44	194	238
	43.14	55.11	52.42
4th	26	81	107
	25.49	23.01	23.57
Total	102	352	454
	100.00	100.00	100.00

imdnew	NC_pos Neg	Pos	Total
1st	21	51	72
	29.17	70.83	100.00
2nd	4	33	37
	10.81	89.19	100.00
3rd	51	187	238
	21.43	78.57	100.00
4th	26	81	107
	24.30	75.70	100.00
Total	102	352	454
	22.47	77.53	100.00

```
Random-effects logistic regression              Number of obs     =        454
Group variable: hh_id                           Number of groups  =        276

Random effects u_i ~ Gaussian                   Obs per group:
                                                              min =          1
                                                              avg =        1.6
                                                              max =          7

Integration method: mvaghermite                 Integration pts.  =         12

                                                Wald chi2(3)      =       3.88
Log likelihood  = -233.38742                    Prob > chi2       =     0.2750

------------------------------------------------------------------------------
```

```
      nc_pos | Odds Ratio   Std. Err.      z    P>|z|     [95% Conf. Interval]
-------------+----------------------------------------------------------------
      imdnew |
        2nd  |   4.341703   3.469031     1.84   0.066     .9068767    20.78606
        3rd  |   1.745245   .8219964     1.18   0.237     .6933421    4.393043
        4th  |   1.303409   .6799066     0.51   0.611     .4688807    3.623257
             |
       _cons |   3.435517   1.479133     2.87   0.004     1.477455    7.988586
-------------+----------------------------------------------------------------
    /lnsig2u |   .7423675   .5314732                     -.2993009    1.784036
-------------+----------------------------------------------------------------
     sigma_u |   1.449449   .3851718                      .8610089    2.440048
         rho |   .3897222    .126405                      .1838995    .6440964
------------------------------------------------------------------------------
Note: Estimates are transformed only in the first equation.
Note: _cons estimates baseline odds (conditional on zero random effects).
LR test of rho=0: chibar2(01) = 11.48                  Prob >= chibar2 = 0.000

Refitting model intpoints() =  8
Refitting model intpoints() = 16

                         Quadrature check

               Fitted       Comparison     Comparison
             quadrature     quadrature     quadrature
             12 points      8 points       16 points
-----------------------------------------------------
Log          -233.38742     -233.39197     -233.38751
likelihood                  -.00454226     -.00008313   Difference
                             .00001946      3.562e-07   Relative difference
-----------------------------------------------------
nc_pos:       1.4682668      1.4699117      1.4683258
1.imdnew                     .00164497      .00005902   Difference
                             .00112035       .0000402   Relative difference
-----------------------------------------------------
nc_pos:       .55689522      .55750266      .55688111
2.imdnew                     .00060744      -.0000141   Difference
                             .00109076     -.00002532   Relative difference
-----------------------------------------------------
nc_pos:        .2649832      .26505685      .26497943
3.imdnew                     .00007366     -3.767e-06   Difference
                             .00027797     -.00001422   Relative difference
-----------------------------------------------------
nc_pos:       1.2341673      1.2348307      1.2341097
   _cons                     .00066342     -.00005758   Difference
                             .00053755     -.00004665   Relative difference
-----------------------------------------------------
/:            .74236747      .74531251      .74224271
 lnsig2u                     .00294504     -.00012476   Difference
                             .00396709     -.00016806   Relative difference
-----------------------------------------------------

           |        vocnew
isolatenew |   Non-VOC        VOC |     Total
-----------+----------------------+----------
        no |        60        263 |       323
           |     58.82      74.72 |     71.15
-----------+----------------------+----------
       yes |        42         89 |       131
           |     41.18      25.28 |     28.85
-----------+----------------------+----------
     Total |       102        352 |       454
           |    100.00     100.00 |    100.00

           |        NC_pos
isolatenew |       Neg        Pos |     Total
-----------+----------------------+----------
        no |        64        259 |       323
           |     19.81      80.19 |    100.00
-----------+----------------------+----------
       yes |        38         93 |       131
           |     29.01      70.99 |    100.00
-----------+----------------------+----------
     Total |       102        352 |       454
           |     22.47      77.53 |    100.00

Random-effects logistic regression              Number of obs     =        454
```

```
Group variable: hh_id                           Number of groups  =        276

Random effects u_i ~ Gaussian                   Obs per group:
                                                              min =          1
                                                              avg =        1.6
                                                              max =          7

Integration method: mvaghermite                 Integration pts.  =         12

                                                Wald chi2(1)      =       3.85
Log likelihood  = -233.47525                    Prob > chi2       =     0.0498

------------------------------------------------------------------------------
      nc_pos | Odds Ratio   Std. Err.      z    P>|z|     [95% Conf. Interval]
-------------+----------------------------------------------------------------
  isolatenew |
        yes  |   .4765333   .1800673    -1.96   0.050      .227222    .9993928
       _cons |   6.907094   2.056209     6.49   0.000     3.853867    12.37924
-------------+----------------------------------------------------------------
    /lnsig2u |   .7975741   .5188084                     -.2192716     1.81442
-------------+----------------------------------------------------------------
     sigma_u |   1.490016   .3865165                      .8961604    2.477401
         rho |   .4029295   .1248135                      .1962153    .6510306
------------------------------------------------------------------------------
Note: Estimates are transformed only in the first equation.
Note: _cons estimates baseline odds (conditional on zero random effects).
LR test of rho=0: chibar2(01) = 12.42                  Prob >= chibar2 = 0.000

Refitting model intpoints() =  8
Refitting model intpoints() = 16

                         Quadrature check

               Fitted       Comparison     Comparison
             quadrature     quadrature     quadrature
             12 points      8 points       16 points
----------------------------------------------------
Log          -233.47525      -233.4815     -233.47542
likelihood                  -.00625457      -.0001701   Difference
                             .00002679      7.285e-07   Relative difference
----------------------------------------------------
nc_pos:      -.74121762     -.74247132     -.74118178
1.isolatenew                 -.0012537      .00003584   Difference
                             .00169141     -.00004836   Relative difference
----------------------------------------------------
nc_pos:        1.932549      1.9344223       1.932442
   _cons                     .00187325     -.00010707   Difference
                             .00096932     -.00005541   Relative difference
----------------------------------------------------
/:            .79757409      .80133424      .79738402
 lnsig2u                     .00376015     -.00019007   Difference
                             .00471448     -.00023831   Relative difference
----------------------------------------------------

close_cont |        vocnew
       act |   Non-VOC        VOC |     Total
-----------+----------------------+----------
         0 |        19         49 |        68
           |     18.63      13.92 |     14.98
-----------+----------------------+----------
         1 |        43        114 |       157
           |     42.16      32.39 |     34.58
-----------+----------------------+----------
         2 |        15         58 |        73
           |     14.71      16.48 |     16.08
-----------+----------------------+----------
         3 |        25        131 |       156
           |     24.51      37.22 |     34.36
-----------+----------------------+----------
     Total |       102        352 |       454
           |    100.00     100.00 |    100.00

close_cont |        NC_pos
       act |       Neg        Pos |     Total
-----------+----------------------+----------
         0 |        24         44 |        68
           |     35.29      64.71 |    100.00
```

```
-----------+----------------------+----------
         1 |        39        118 |       157 
           |     24.84      75.16 |    100.00 
-----------+----------------------+----------
         2 |        14         59 |        73 
           |     19.18      80.82 |    100.00 
-----------+----------------------+----------
         3 |        25        131 |       156 
           |     16.03      83.97 |    100.00 
-----------+----------------------+----------
     Total |       102        352 |       454 
           |     22.47      77.53 |    100.00 

Random-effects logistic regression              Number of obs     =        454
Group variable: hh_id                           Number of groups  =        276

Random effects u_i ~ Gaussian                   Obs per group:
                                                              min =          1
                                                              avg =        1.6
                                                              max =          7

Integration method: mvaghermite                 Integration pts.  =         12

                                                Wald chi2(3)      =       8.58
Log likelihood  = -230.63371                    Prob > chi2       =     0.0354

-------------------------------------------------------------------------------
       nc_pos | Odds Ratio   Std. Err.      z    P>|z|     [95% Conf. Interval]
--------------+----------------------------------------------------------------
close_contact |
           1  |   1.931561   .9047636     1.41   0.160     .7712526    4.837492
           2  |   3.411578   1.947697     2.15   0.032     1.114288    10.44511
           3  |   3.860246   1.932494     2.70   0.007     1.447076    10.29766
              |
        _cons |   2.334521   .9437257     2.10   0.036     1.057064     5.15578
--------------+----------------------------------------------------------------
     /lnsig2u |   .8614943   .5371987                     -.1913958    1.914384
--------------+----------------------------------------------------------------
      sigma_u |   1.538407    .413215                      .9087385    2.604374
          rho |    .418398   .1307225                       .200649    .6733854
-------------------------------------------------------------------------------
Note: Estimates are transformed only in the first equation.
Note: _cons estimates baseline odds (conditional on zero random effects).
LR test of rho=0: chibar2(01) = 11.73                  Prob >= chibar2 = 0.000

Refitting model intpoints() =  8
Refitting model intpoints() = 16

                         Quadrature check

               Fitted      Comparison     Comparison
             quadrature    quadrature     quadrature
             12 points     8 points       16 points
-----------------------------------------------------
Log          -230.63371    -230.64074     -230.63395
likelihood                 -.00703513     -.00023769   Difference
                             .0000305      1.031e-06   Relative difference
-----------------------------------------------------
nc_pos:       .65832848     .65941462      .65827697
1.close_co~t                .00108614     -.00005151   Difference
                            .00164985     -.00007824   Relative difference
-----------------------------------------------------
nc_pos:       1.2271749     1.2296356      1.2270703
2.close_co~t                .00246073     -.00010455   Difference
                             .0020052      -.0000852   Relative difference
-----------------------------------------------------
nc_pos:        1.350731     1.3529793       1.350674
3.close_co~t                .00224837     -.00005701   Difference
                            .00166456      -.0000422   Relative difference
-----------------------------------------------------
nc_pos:       .84780661      .8483896       .8477465
   _cons                    .00058299     -.00006011   Difference
                            .00068765      -.0000709   Relative difference
-----------------------------------------------------
/:            .86149431     .86668334      .86127062
 lnsig2u                    .00518903     -.00022369   Difference
                            .00602328     -.00025966   Relative difference
```

```
--------------------------------------------------

 RECODE of |
diffpcrser |        vocnew
         o |   Non-VOC        VOC |     Total
-----------+----------------------+----------
      <100 |         2        102 |       104 
           |      1.96      28.98 |     22.91 
-----------+----------------------+----------
   100-150 |        58        148 |       206 
           |     56.86      42.05 |     45.37 
-----------+----------------------+----------
      >150 |        42        102 |       144 
           |     41.18      28.98 |     31.72 
-----------+----------------------+----------
     Total |       102        352 |       454 
           |    100.00     100.00 |    100.00 

 RECODE of |
diffpcrser |        NC_pos
         o |       Neg        Pos |     Total
-----------+----------------------+----------
      <100 |        13         91 |       104 
           |     12.50      87.50 |    100.00 
-----------+----------------------+----------
   100-150 |        47        159 |       206 
           |     22.82      77.18 |    100.00 
-----------+----------------------+----------
      >150 |        42        102 |       144 
           |     29.17      70.83 |    100.00 
-----------+----------------------+----------
     Total |       102        352 |       454 
           |     22.47      77.53 |    100.00 

Random-effects logistic regression              Number of obs     =        454
Group variable: hh_id                           Number of groups  =        276

Random effects u_i ~ Gaussian                   Obs per group:
                                                              min =          1
                                                              avg =        1.6
                                                              max =          7

Integration method: mvaghermite                 Integration pts.  =         12

                                                Wald chi2(2)      =       7.05
Log likelihood  = -231.48092                    Prob > chi2       =     0.0295

------------------------------------------------------------------------------
          nc_pos | Odds Ratio   Std. Err.      z    P>|z|     [95% Conf. Interval]
-----------------+------------------------------------------------------------
diffpcrsero_cat3 |
        100-150  |   .3882039   .1874793    -1.96   0.050     .1506539    1.000321
           >150  |   .2607014   .1320531    -2.65   0.008     .0966016    .7035623
                 |
           _cons |   13.26599   6.569921     5.22   0.000     5.025576    35.01816
-----------------+------------------------------------------------------------
        /lnsig2u |   .6964202   .5368976                     -.3558797     1.74872
-----------------+------------------------------------------------------------
         sigma_u |    1.41653   .3802657                      .8369928    2.397341
             rho |   .3788512   .1263443                      .1755595    .6359602
------------------------------------------------------------------------------
Note: Estimates are transformed only in the first equation.
Note: _cons estimates baseline odds (conditional on zero random effects).
LR test of rho=0: chibar2(01) = 10.51                 Prob >= chibar2 = 0.001

Refitting model intpoints() =  8
Refitting model intpoints() = 16

                         Quadrature check

             Fitted      Comparison     Comparison
             quadrature  quadrature     quadrature
             12 points   8 points       16 points
-----------------------------------------------------
Log          -231.48092  -231.48372     -231.48096
likelihood               -.00279762     -.00003857   Difference
                          .00001209      1.666e-07   Relative difference
```

```
-----------------------------------------------------
nc_pos:      -.94622459     -.94749653     -.94629708
2.diffpcrs~3                -.00127194     -.00007249   Difference
                             .00134423      .00007661   Relative difference
-----------------------------------------------------
nc_pos:      -1.3443796     -1.3460817     -1.3444416
3.diffpcrs~3                -.00170211     -.00006202   Difference
                             .0012661       .00004613   Relative difference
-----------------------------------------------------
nc_pos:       2.5852035      2.5874489      2.5852545
   _cons                     .00224546       .000051    Difference
                             .00086858      .00001973   Relative difference
-----------------------------------------------------
/:            .69642019      .6992171       .69641754
 lnsig2u                     .00279692     -2.647e-06   Difference
                             .00401613     -3.802e-06   Relative difference
-----------------------------------------------------
```

```
 RECODE of |        vocnew
    dayshh |   Non-VOC        VOC |     Total
-----------+----------------------+----------
         0 |        23         33 |        56
           |     22.55       9.38 |     12.33
-----------+----------------------+----------
      1-3d |        22         61 |        83
           |     21.57      17.33 |     18.28
-----------+----------------------+----------
      4-7d |        26        111 |       137
           |     25.49      31.53 |     30.18
-----------+----------------------+----------
       >7d |        31        147 |       178
           |     30.39      41.76 |     39.21
-----------+----------------------+----------
     Total |       102        352 |       454
           |    100.00     100.00 |    100.00
```

```
 RECODE of |        NC_pos
    dayshh |       Neg        Pos |     Total
-----------+----------------------+----------
         0 |        19         37 |        56
           |     33.93      66.07 |    100.00
-----------+----------------------+----------
      1-3d |        24         59 |        83
           |     28.92      71.08 |    100.00
-----------+----------------------+----------
      4-7d |        29        108 |       137
           |     21.17      78.83 |    100.00
-----------+----------------------+----------
       >7d |        30        148 |       178
           |     16.85      83.15 |    100.00
-----------+----------------------+----------
     Total |       102        352 |       454
           |     22.47      77.53 |    100.00
```

```
Random-effects logistic regression              Number of obs     =        454
Group variable: hh_id                           Number of groups  =        276

Random effects u_i ~ Gaussian                   Obs per group:
                                                              min =          1
                                                              avg =        1.6
                                                              max =          7

Integration method: mvaghermite                 Integration pts.  =         12

                                                Wald chi2(3)      =       7.55
Log likelihood  = -231.10402                    Prob > chi2       =     0.0563

------------------------------------------------------------------------------
      nc_pos | Odds Ratio   Std. Err.      z    P>|z|     [95% Conf. Interval]
-------------+----------------------------------------------------------------
  dayshh_cat |
       1-3d  |   1.323575   .7179555     0.52   0.605     .4571155    3.832405
       4-7d  |   2.507044    1.34648     1.71   0.087     .8749831    7.183302
        >7d  |   3.773342   2.076606     2.41   0.016     1.283155    11.09617
             |
       _cons |   2.460671    1.10357     2.01   0.045     1.021654    5.926566
-------------+----------------------------------------------------------------
```

```
    /lnsig2u |   .8381753   .5306845                     -.2019473    1.878298
-------------+----------------------------------------------------------------
     sigma_u |   1.520574   .4034724                      .9039568    2.557804
         rho |   .4127346   .1286298                       .198962    .6653995
------------------------------------------------------------------------------
Note: Estimates are transformed only in the first equation.
Note: _cons estimates baseline odds (conditional on zero random effects).
LR test of rho=0: chibar2(01) = 12.27                  Prob >= chibar2 = 0.000

Refitting model intpoints() =  8
Refitting model intpoints() = 16

                         Quadrature check

               Fitted      Comparison     Comparison
             quadrature    quadrature     quadrature
             12 points     8 points       16 points
-----------------------------------------------------
Log          -231.10402    -231.11028     -231.10422
likelihood                 -.00625626     -.00019967   Difference
                            .00002707      8.640e-07   Relative difference
-----------------------------------------------------
nc_pos:       .28033671     .28051364      .28033181
1.dayshh_cat                .00017693     -4.897e-06   Difference
                            .00063115     -.00001747   Relative difference
-----------------------------------------------------
nc_pos:       .91910423     .92072542      .91905128
2.dayshh_cat                .00162119     -.00005296   Difference
                            .00176388     -.00005762   Relative difference
-----------------------------------------------------
nc_pos:       1.3279611     1.3304537       1.327941
3.dayshh_cat                .00249265     -.00002002   Difference
                            .00187705     -.00001508   Relative difference
-----------------------------------------------------
nc_pos:       .90043412      .9009076      .90038752
   _cons                    .00047348      -.0000466   Difference
                            .00052583     -.00005175   Relative difference
-----------------------------------------------------
/:            .83817526     .84286857      .83803938
 lnsig2u                    .00469331     -.00013589   Difference
                            .00559943     -.00016212   Relative difference
-----------------------------------------------------
```

3. Summary of VOC and nc_pos - unadjusted

```
.
. tab vocnew nc_pos, row

           |        NC_pos
    vocnew |       Neg        Pos |     Total
-----------+----------------------+----------
   Non-VOC |        39         63 |       102 
           |     38.24      61.76 |    100.00 
-----------+----------------------+----------
       VOC |        63        289 |       352 
           |     17.90      82.10 |    100.00 
-----------+----------------------+----------
     Total |       102        352 |       454 
           |     22.47      77.53 |    100.00 


. xtlogit nc_pos i.vocnew, re or nolog/* Random effects logistic. [3.54] NB: rho is large
(0.33) so numerical approx should be checked*/

Random-effects logistic regression              Number of obs     =        454
Group variable: hh_id                           Number of groups  =        276

Random effects u_i ~ Gaussian                   Obs per group:
                                                              min =          1
                                                              avg =        1.6
                                                              max =          7

Integration method: mvaghermite                 Integration pts.  =         12

                                                Wald chi2(1)      =      11.41
Log likelihood  = -229.46051                    Prob > chi2       =     0.0007
------------------------------------------------------------------------------
      nc_pos | Odds Ratio   Std. Err.      z    P>|z|     [95% Conf. Interval]
-------------+----------------------------------------------------------------
      vocnew |
        VOC  |   3.542754    1.32668     3.38   0.001     1.700536    7.380679
       _cons |   1.943125   .6121677     2.11   0.035     1.047938    3.603012
-------------+----------------------------------------------------------------
    /lnsig2u |   .4897778   .5991911                     -.684615    1.664171
-------------+----------------------------------------------------------------
     sigma_u |   1.277479   .3827271                     .7101298    2.298106
         rho |   .3315751   .1328005                     .1329109      .61617
------------------------------------------------------------------------------
Note: Estimates are transformed only in the first equation.
Note: _cons estimates baseline odds (conditional on zero random effects).
LR test of rho=0: chibar2(01) = 7.55                   Prob >= chibar2 = 0.003

. quadchk, nooutput

Refitting model intpoints() =  8
Refitting model intpoints() = 16

                         Quadrature check

              Fitted       Comparison     Comparison
             quadrature    quadrature     quadrature
             12 points     8 points       16 points
-----------------------------------------------------
Log          -229.46051    -229.46195     -229.46057
likelihood                 -.00143828     -.00006136   Difference
                            6.268e-06      2.674e-07   Relative difference
-----------------------------------------------------
nc_pos:       1.2649045     1.2655141       1.264907
1.vocnew                    .00060965      2.516e-06   Difference
                            .00048197      1.989e-06   Relative difference
-----------------------------------------------------
nc_pos:       .66429728     .66454057      .66430144
   _cons                    .00024329      4.165e-06   Difference
                            .00036624      6.270e-06   Relative difference
-----------------------------------------------------
/:            .48977785     .49191591      .48980178
 lnsig2u                    .00213807      .00002394   Difference
                            .00436538      .00004887   Relative difference
-----------------------------------------------------
```

4. Summary of VOC and nc_pos – adjusted (with continuous covariates grouped – diffpcrsero [3 levels]; dayshh [4 levels]; imd [4 levels])

```
. xtlogit nc_pos i.vocnew i.sexnew agenew i.diffpcrsero_cat3 i.dayshh_cat i.imdnew
i.close_contact, re or  nolog

Random-effects logistic regression              Number of obs     =        454
Group variable: hh_id                           Number of groups  =        276

Random effects u_i ~ Gaussian                   Obs per group:
                                                              min =          1
                                                              avg =        1.6
                                                              max =          7

Integration method: mvaghermite                 Integration pts.  =         12

                                                Wald chi2(14)     =      20.73
Log likelihood  = -220.37474                    Prob > chi2       =     0.1088

------------------------------------------------------------------------------
          nc_pos | Odds Ratio   Std. Err.      z    P>|z|     [95% Conf. Interval]
-----------------+------------------------------------------------------------
          vocnew |
            VOC  |   2.394783   .9624276     2.17   0.030     1.089384     5.26443
                 |
          sexnew |
          female |   .8586828     .25708    -0.51   0.611     .4775211    1.544091
          agenew |   1.004246   .0075533     0.56   0.573     .9895503     1.01916
                 |
diffpcrsero_cat3 |
        100-150  |   .5220589   .2544353    -1.33   0.182     .2008493    1.356965
           >150  |   .3408856    .174178    -2.11   0.035     .1252221    .9279754
                 |
      dayshh_cat |
           1-3d  |   .9645288   .5266307    -0.07   0.947     .3307965    2.812351
           4-7d  |   1.386087   .7818336     0.58   0.563     .4588419    4.187141
            >7d  |   1.892842   1.070006     1.13   0.259      .625084    5.731793
                 |
          imdnew |
            2nd  |   2.419628   1.946274     1.10   0.272     .5001131    11.70655
            3rd  |   1.105704     .541181    0.21   0.837     .4236655    2.885725
            4th  |   .9940233   .5347686    -0.01   0.991      .346309    2.853181
                 |
   close_contact |
              1  |   1.538946   .7458413     0.89   0.374     .5952419    3.978811
              2  |   2.105478   1.263663     1.24   0.215     .6493481    6.826903
              3  |   2.706174   1.459996     1.85   0.065     .9399994    7.790832
                 |
           _cons |    1.66391   1.344699     0.63   0.529     .3413708    8.110235
-----------------+------------------------------------------------------------
        /lnsig2u |     .50901   .6437654                     -.752747    1.770767
-----------------+------------------------------------------------------------
         sigma_u |   1.289823   .4151717                     .6863459    2.423914
             rho |   .3358513   .1435952                     .1252535    .6410489
------------------------------------------------------------------------------
Note: Estimates are transformed only in the first equation.
Note: _cons estimates baseline odds (conditional on zero random effects).
LR test of rho=0: chibar2(01) = 6.10                   Prob >= chibar2 = 0.007
```

5. Summary of VOC and nc_pos – adjusted (with continuous covariates + age assessed using fractional polynomals); no adjustment for close_contact

```
.
. * fractional polynomials for continuous terms for agenewm, diffpcrsero and dayshh
. mfp: xtlogit nc_pos vocnew agenew diffpcrsero dayshh  imd_rank, re or  nolog

Deviance for model with all terms untransformed =   451.141, 454 observations

[vocnew included with 1 df in model]

diffpcrsero  lin.   FP2     451.141     0.281  0.964    1         0 3
             Final          451.141                     1

dayshh       lin.   FP2     451.141     6.180  0.103    1         1 2
             Final          451.141                     1

agenew       lin.   FP2     451.141     0.797  0.850    1         3 3
             Final          451.141                     1

imd_rank     lin.   FP2     451.141     0.759  0.859    1         -2 -1
             Final          451.141                     1


Fractional polynomial fitting algorithm converged after 1 cycle.

Transformations of covariates:

-> gen double Iagen__1 = agenew-41.10352423 if e(sample)
-> gen double Idiff__1 = diffpcrsero-130.7400881 if e(sample)
-> gen double Idays__1 = dayshh-8.372246696 if e(sample)
-> gen double Iimd___1 = imd_rank-11221.59251 if e(sample)

Final multivariable fractional polynomial model for nc_pos
--------------------------------------------------------------------
    Variable |    -----Initial-----          -----Final-----
             |   df     Select   Alpha    Status    df    Powers
-------------+------------------------------------------------------
      vocnew |    1     1.0000   0.0500     in       1     1
      agenew |    4     1.0000   0.0500     in       1     1
 diffpcrsero |    4     1.0000   0.0500     in       1     1
      dayshh |    4     1.0000   0.0500     in       1     1
    imd_rank |    4     1.0000   0.0500     in       1     1
--------------------------------------------------------------------

Random-effects logistic regression               Number of obs     =        454
Group variable: hh_id                            Number of groups  =        276

Random effects u_i ~ Gaussian                    Obs per group:
                                                              min =          1
                                                              avg =        1.6
                                                              max =          7

Integration method: mvaghermite                  Integration pts.  =         12

                                                 Wald chi2(5)      =      16.47
Log likelihood  = -225.57054                     Prob > chi2       =     0.0056

------------------------------------------------------------------------------
      nc_pos | Odds Ratio   Std. Err.      z    P>|z|     [95% Conf. Interval]
-------------+----------------------------------------------------------------
      vocnew |   2.706961   1.058336     2.55   0.011     1.258025    5.824717
    Iagen__1 |   1.009618   .0069873     1.38   0.167     .9960159    1.023406
    Idiff__1 |    .991264    .004792    -1.82   0.070     .9819163    1.000701
    Idays__1 |   1.033742   .0217829     1.57   0.115     .9919178     1.07733
    Iimd___1 |   .9999948   .0000209    -0.25   0.804     .9999538    1.000036
       _cons |   2.426699    .821718     2.62   0.009     1.249643    4.712441
-------------+----------------------------------------------------------------
    /lnsig2u |   .4524772   .6260598                     -.7745774    1.679532
-------------+----------------------------------------------------------------
     sigma_u |   1.253875   .3925003                      .6788951    2.315825
         rho |   .3233606    .136981                      .1228812    .6197964
------------------------------------------------------------------------------
```

```
Note: Estimates are transformed only in the first equation.
Note: _cons estimates baseline odds (conditional on zero random effects).
LR test of rho=0: chibar2(01) = 6.77                   Prob >= chibar2 = 0.005
Deviance:  451.141.

.
.
```

6. Summary of VOC and nc_pos – adjusted (with continuous covariates + age assessed using fractional polynomals); plus adjustment for close_contact (assumed as factors)

```
. mfp, df(agenew diffpcrsero dayshh imd_rank:4): xtlogit nc_pos vocnew sexnew agenew
diffpcrsero dayshh imd_rank close_contacta close_contactb close_contactc, re or  nolog

Deviance for model with all terms untransformed =   445.167, 454 observations

[vocnew included with 1 df in model]

[close_contactc included with 1 df in model]

diffpcrsero  lin.   FP2     445.167     0.180  0.981    1         2 3
             Final          445.167                     1

[close_contactb included with 1 df in model]

[close_contacta included with 1 df in model]

dayshh       lin.   FP2     445.167     3.877  0.275    1         1 2
             Final          445.167                     1

agenew       lin.   FP2     445.167     0.777  0.855    1         -2 2
             Final          445.167                     1

[sexnew included with 1 df in model]

imd_rank     lin.   FP2     445.167     0.810  0.847    1         -2 -1
             Final          445.167                     1


Fractional polynomial fitting algorithm converged after 1 cycle.

Transformations of covariates:

-> gen double Iagen__1 = agenew-41.10352423 if e(sample)
-> gen double Idiff__1 = diffpcrsero-130.7400881 if e(sample)
-> gen double Idays__1 = dayshh-8.372246696 if e(sample)
-> gen double Iimd___1 = imd_rank-11221.59251 if e(sample)

Final multivariable fractional polynomial model for nc_pos
--------------------------------------------------------------------
    Variable |    -----Initial-----          -----Final-----
             |   df     Select   Alpha    Status    df    Powers
-------------+------------------------------------------------------
      vocnew |    1     1.0000   0.0500     in       1     1
      sexnew |    1     1.0000   0.0500     in       1     1
      agenew |    4     1.0000   0.0500     in       1     1
 diffpcrsero |    4     1.0000   0.0500     in       1     1
      dayshh |    4     1.0000   0.0500     in       1     1
    imd_rank |    4     1.0000   0.0500     in       1     1
close_con... |    1     1.0000   0.0500     in       1     1
close_con... |    1     1.0000   0.0500     in       1     1
close_con... |    1     1.0000   0.0500     in       1     1
--------------------------------------------------------------------

Random-effects logistic regression              Number of obs     =        454
Group variable: hh_id                           Number of groups  =        276

Random effects u_i ~ Gaussian                   Obs per group:
                                                              min =          1
                                                              avg =        1.6
                                                              max =          7
```

```
Integration method: mvaghermite              Integration pts.  =         12

                                             Wald chi2(9)      =      19.34
Log likelihood  = -222.58337                 Prob > chi2       =     0.0224

--------------------------------------------------------------------------------
        nc_pos | Odds Ratio   Std. Err.      z    P>|z|     [95% Conf. Interval]
---------------+----------------------------------------------------------------
        vocnew |   2.541672   1.012601     2.34   0.019     1.164129    5.549293
        sexnew |   .8790088   .2592811    -0.44   0.662     .4930767    1.567011
      Iagen__1 |   1.004305    .007453     0.58   0.563     .9898034     1.01902
      Idiff__1 |   .9902699   .0049169    -1.97   0.049     .9806796    .9999539
      Idays__1 |   1.021089   .0220517     0.97   0.334     .9787699    1.065237
      Iimd___1 |   1.000001   .0000218     0.03   0.974     .9999581    1.000043
 close_contacta |   1.805578   .8254464     1.29   0.196     .7370162    4.423392
 close_contactb |   2.513999   1.409449     1.64   0.100     .8378131    7.543675
 close_contactc |   3.174559   1.607752     2.28   0.023     1.176504    8.565912
         _cons |   1.320507   .6578477     0.56   0.577     .4973826    3.505829
---------------+----------------------------------------------------------------
      /lnsig2u |   .4770832   .6460376                     -.7891273    1.743294
---------------+----------------------------------------------------------------
       sigma_u |   1.269397   .4100389                      .6739741    2.390845
           rho |   .3287676   .1425672                      .1213215    .6347029
--------------------------------------------------------------------------------
Note: Estimates are transformed only in the first equation.
Note: _cons estimates baseline odds (conditional on zero random effects).
LR test of rho=0: chibar2(01) = 6.15                   Prob >= chibar2 = 0.007
Deviance:  445.167.
```

7. Summary of VOC and nc_pos – adjusted (with continuous covariates + age assessed using fractional polynomals); adjustment for close_contact (assuming quantitative)

```
. mfp, df(agenew diffpcrsero dayshh imd_rank close_contact:4): xtlogit nc_pos vocnew sexnew
agenew diffpcrsero dayshh imd_rank close_contact, re or  nolog

Deviance for model with all terms untransformed =   445.533, 454 observations

[vocnew included with 1 df in model]

close_con... lin.   FP2     445.533     0.366  0.947    1          -2 0
             Final          445.533                     1

diffpcrsero  lin.   FP2     445.533     0.198  0.978    1          1 3
             Final          445.533                     1

dayshh       lin.   FP2     445.533     4.148  0.246    1          1 2
             Final          445.533                     1

agenew       lin.   FP2     445.533     0.881  0.830    1          -1 1
             Final          445.533                     1

[sexnew included with 1 df in model]

imd_rank     lin.   FP2     445.533     0.717  0.869    1          -2 -1
             Final          445.533                     1


Fractional polynomial fitting algorithm converged after 1 cycle.

Transformations of covariates:

-> gen double Iagen__1 = agenew-41.10352423 if e(sample)
-> gen double Idiff__1 = diffpcrsero-130.7400881 if e(sample)
-> gen double Idays__1 = dayshh-8.372246696 if e(sample)
-> gen double Iimd___1 = imd_rank-11221.59251 if e(sample)
-> gen double Iclos__1 = close_contact-1.698237885 if e(sample)

Final multivariable fractional polynomial model for nc_pos
--------------------------------------------------------------------
    Variable |    -----Initial-----          -----Final-----
```

```
              |   df     Select   Alpha    Status    df    Powers
--------------+------------------------------------------------------
       vocnew |    1     1.0000   0.0500     in       1     1
       sexnew |    1     1.0000   0.0500     in       1     1
       agenew |    4     1.0000   0.0500     in       1     1
  diffpcrsero |    4     1.0000   0.0500     in       1     1
       dayshh |    4     1.0000   0.0500     in       1     1
     imd_rank |    4     1.0000   0.0500     in       1     1
close_con...  |    4     1.0000   0.0500     in       1     1
---------------------------------------------------------------------

Random-effects logistic regression              Number of obs     =        454
Group variable: hh_id                           Number of groups  =        276

Random effects u_i ~ Gaussian                   Obs per group:
                                                              min =          1
                                                              avg =        1.6
                                                              max =          7

Integration method: mvaghermite                 Integration pts.  =         12

                                                Wald chi2(7)      =      19.48
Log likelihood  = -222.76656                    Prob > chi2       =     0.0068

------------------------------------------------------------------------------
      nc_pos | Odds Ratio   Std. Err.      z    P>|z|     [95% Conf. Interval]
-------------+----------------------------------------------------------------
      vocnew |   2.485695   .9772936     2.32   0.021     1.150227    5.371707
      sexnew |   .8820909   .2581135    -0.43   0.668      .497094    1.565266
    Iagen__1 |    1.00412   .0072218     0.57   0.568     .9900645    1.018374
    Idiff__1 |   .9901183   .0048778    -2.02   0.044      .980604    .9997249
    Idays__1 |   1.022191   .0219288     1.02   0.306     .9801028    1.066087
    Iimd___1 |   .9999992   .0000212    -0.04   0.971     .9999577    1.000041
    Iclos__1 |   1.422322   .2195464     2.28   0.022     1.051013    1.924808
       _cons |    2.82485   1.088493     2.69   0.007     1.327406    6.011556
-------------+----------------------------------------------------------------
    /lnsig2u |   .4492293   .6411026                     -.8073086    1.705767
-------------+----------------------------------------------------------------
     sigma_u |    1.25184    .401279                       .667875    2.346403
         rho |   .3226504   .1401111                      .1193967    .6259591
------------------------------------------------------------------------------
Note: Estimates are transformed only in the first equation.
Note: _cons estimates baseline odds (conditional on zero random effects).
LR test of rho=0: chibar2(01) = 6.14                   Prob >= chibar2 = 0.007
Deviance:  445.533.
```

8. Summary of VOC and nc_pos

	% NC_pos (n/N)	Unadjusted OR (95% CI)	P-value	Adjusted OR*(95% CI)	P-value
Non-VOC	62% (63/102)				
VOC	82% (289/352)	3.5 (1.7-7.4)	0.001	2.5 (1.2-5.4)	0.02

* adjusted for sex, age (years; linear), time since pcr_date date to date of HH serology (days; linear), infectious index and HHC in contact (days; linear), IMD (rank; linear), close contacts (4 levels assuming a linear trend)

9. Summary of potential confounders with infection

```
. ********** new outcome : infected
.
. foreach var in sexnew imdnew isolatenew close_contact diffpcrsero_cat3 dayshh_cat {
  2.
.         tab `var' infected, row nokey
  3.         xtlogit infected i.`var', re or nolog
  4.         quadchk, nooutput
  5. }

           |       INFECTED
    sexnew |         0          1 |     Total
-----------+----------------------+----------
      male |        28        165 |       193 
           |     14.51      85.49 |    100.00 
-----------+----------------------+----------
    female |        42        219 |       261 
           |     16.09      83.91 |    100.00 
-----------+----------------------+----------
     Total |        70        384 |       454 
           |     15.42      84.58 |    100.00 

Random-effects logistic regression              Number of obs     =        454
Group variable: hh_id                           Number of groups  =        276

Random effects u_i ~ Gaussian                   Obs per group:
                                                              min =          1
                                                              avg =        1.6
                                                              max =          7

Integration method: mvaghermite                 Integration pts.  =         12

                                                Wald chi2(1)      =       0.47
Log likelihood  = -189.12006                    Prob > chi2       =     0.4947

------------------------------------------------------------------------------
    infected | Odds Ratio   Std. Err.      z    P>|z|     [95% Conf. Interval]
-------------+----------------------------------------------------------------
      sexnew |
     female  |     .77924   .2846242    -0.68   0.495     .3808575    1.594337
       _cons |   14.60683   7.184001     5.45   0.000     5.570756    38.29993
-------------+----------------------------------------------------------------
    /lnsig2u |    1.26862   .5693602                      .1526943    2.384545
-------------+----------------------------------------------------------------
     sigma_u |    1.88572   .5368271                      1.079337     3.29456
         rho |   .5194333    .142125                      .2615065    .7674018
------------------------------------------------------------------------------
Note: Estimates are transformed only in the first equation.
Note: _cons estimates baseline odds (conditional on zero random effects).
LR test of rho=0: chibar2(01) = 11.89                  Prob >= chibar2 = 0.000

Refitting model intpoints() =  8
Refitting model intpoints() = 16

                         Quadrature check

               Fitted       Comparison     Comparison
             quadrature     quadrature     quadrature
             12 points      8 points       16 points
-----------------------------------------------------
Log          -189.12006     -189.1508      -189.12425
likelihood                 -.03074183     -.00419064   Difference
                            .00016255      .00002216   Relative difference
-----------------------------------------------------
infected:    -.24943615     -.25343139     -.24967418
1.sexnew                   -.00399523     -.00023803   Difference
                            .01601705      .00095427   Relative difference
-----------------------------------------------------
infected:     2.6814894      2.7226579      2.6817572
   _cons                    .04116845      .00026779   Difference
                            .01535283      .00009987   Relative difference
-----------------------------------------------------
/:            1.2686198      1.3201621      1.2688845
 lnsig2u                    .05154226      .00026465   Difference
                            .04062861      .00020862   Relative difference
```

```
-----------------------------------------------------

           |       INFECTED
    imdnew |         0          1 |     Total
-----------+----------------------+----------
       1st |        17         55 |        72 
           |     23.61      76.39 |    100.00 
-----------+----------------------+----------
       2nd |         3         34 |        37 
           |      8.11      91.89 |    100.00 
-----------+----------------------+----------
       3rd |        32        206 |       238 
           |     13.45      86.55 |    100.00 
-----------+----------------------+----------
       4th |        18         89 |       107 
           |     16.82      83.18 |    100.00 
-----------+----------------------+----------
     Total |        70        384 |       454 
           |     15.42      84.58 |    100.00 

Random-effects logistic regression              Number of obs     =        454
Group variable: hh_id                           Number of groups  =        276

Random effects u_i ~ Gaussian                   Obs per group:
                                                              min =          1
                                                              avg =        1.6
                                                              max =          7

Integration method: mvaghermite                 Integration pts.  =         12

                                                Wald chi2(3)      =       4.20
Log likelihood  = -187.03158                    Prob > chi2       =     0.2406

------------------------------------------------------------------------------
    infected | Odds Ratio   Std. Err.      z    P>|z|     [95% Conf. Interval]
-------------+----------------------------------------------------------------
      imdnew |
        2nd  |   5.122131   4.962827     1.69   0.092     .7668712    34.21204
        3rd  |   2.707812   1.565596     1.72   0.085     .8719126    8.409381
        4th  |   1.729985   1.081287     0.88   0.381     .5081808    5.889335
             |
       _cons |   5.625091   3.024681     3.21   0.001     1.960776    16.13731
-------------+----------------------------------------------------------------
    /lnsig2u |   1.187346   .5951741                      .0208259    2.353865
-------------+----------------------------------------------------------------
     sigma_u |   1.810626   .5388189                      1.010467    3.244407
         rho |   .4991245   .1487931                      .2368511    .7618807
------------------------------------------------------------------------------
Note: Estimates are transformed only in the first equation.
Note: _cons estimates baseline odds (conditional on zero random effects).
LR test of rho=0: chibar2(01) = 10.33                  Prob >= chibar2 = 0.001

Refitting model intpoints() =  8
Refitting model intpoints() = 16

                         Quadrature check

               Fitted       Comparison     Comparison
             quadrature     quadrature     quadrature
             12 points      8 points       16 points
-----------------------------------------------------
Log          -187.03158     -187.04944       -187.034
likelihood                  -.01786185     -.00242075   Difference
                              .0000955      .00001294   Relative difference
-----------------------------------------------------
infected:     1.6335705      1.6524734      1.6347075
1.imdnew                     .01890289      .00113693   Difference
                             .01157152      .00069598   Relative difference
-----------------------------------------------------
infected:      .9961409      1.0105823      .99658433
2.imdnew                     .01444139      .00044343   Difference
                             .01449733      .00044515   Relative difference
-----------------------------------------------------
infected:     .54811249      .55427786      .54816655
3.imdnew                     .00616537      .00005405   Difference
                             .01124837      .00009862   Relative difference
-----------------------------------------------------
```

```
infected:      1.727237      1.7452364      1.726907
   _cons                     .01799937    -.00033002   Difference
                              .0104209    -.00019107   Relative difference
-----------------------------------------------------
/:            1.1873457      1.2278071      1.1872109
 lnsig2u                     .04046143    -.00013471   Difference
                             .03407721    -.00011345   Relative difference
-----------------------------------------------------

           |       INFECTED
isolatenew |         0          1 |     Total
-----------+----------------------+----------
        no |        40        283 |       323
           |     12.38      87.62 |    100.00
-----------+----------------------+----------
       yes |        30        101 |       131
           |     22.90      77.10 |    100.00
-----------+----------------------+----------
     Total |        70        384 |       454
           |     15.42      84.58 |    100.00

Random-effects logistic regression              Number of obs     =        454
Group variable: hh_id                           Number of groups  =        276

Random effects u_i ~ Gaussian                   Obs per group:
                                                              min =          1
                                                              avg =        1.6
                                                              max =          7

Integration method: mvaghermite                 Integration pts.  =         12

                                                Wald chi2(1)      =       4.94
Log likelihood  = -186.66877                    Prob > chi2       =     0.0262

------------------------------------------------------------------------------
    infected | Odds Ratio   Std. Err.      z    P>|z|     [95% Conf. Interval]
-------------+----------------------------------------------------------------
  isolatenew |
        yes  |    .364505   .1655033    -2.22   0.026      .149698    .8875463
       _cons |   15.93961   7.471103     5.91   0.000     6.360752    39.94358
-------------+----------------------------------------------------------------
    /lnsig2u |   1.142566   .6111612                     -.0552882     2.34042
-------------+----------------------------------------------------------------
     sigma_u |   1.770537   .5410418                      .9727345    3.222669
         rho |   .4879319   .1527013                      .2233698    .7594328
------------------------------------------------------------------------------
Note: Estimates are transformed only in the first equation.
Note: _cons estimates baseline odds (conditional on zero random effects).
LR test of rho=0: chibar2(01) = 9.57                   Prob >= chibar2 = 0.001

Refitting model intpoints() =  8
Refitting model intpoints() = 16

                         Quadrature check

              Fitted       Comparison     Comparison
            quadrature     quadrature     quadrature
            12 points      8 points       16 points
-----------------------------------------------------
Log         -186.66877      -186.6835     -186.67081
likelihood                 -.01473135     -.0020341   Difference
                            .00007892      .0000109   Relative difference
-----------------------------------------------------
infected:   -1.0092149     -1.0216711     -1.0100267
1.isolatenew               -.01245616    -.00081181   Difference
                            .01234242      .0008044   Relative difference
-----------------------------------------------------
infected:    2.7688072      2.7982461      2.770061
   _cons                    .02943888     .00125377   Difference
                            .01063233     .00045282   Relative difference
-----------------------------------------------------
/:           1.1425657      1.1812145     1.1441298
 lnsig2u                    .03864879      .0015641   Difference
                            .03382632     .00136894   Relative difference
-----------------------------------------------------
```

```
close_cont |       INFECTED
       act |         0          1 |     Total
-----------+----------------------+----------
         0 |        17         51 |        68
           |     25.00      75.00 |    100.00
-----------+----------------------+----------
         1 |        21        136 |       157
           |     13.38      86.62 |    100.00
-----------+----------------------+----------
         2 |        11         62 |        73
           |     15.07      84.93 |    100.00
-----------+----------------------+----------
         3 |        21        135 |       156
           |     13.46      86.54 |    100.00
-----------+----------------------+----------
     Total |        70        384 |       454
           |     15.42      84.58 |    100.00

Random-effects logistic regression              Number of obs     =        454
Group variable: hh_id                           Number of groups  =        276

Random effects u_i ~ Gaussian                   Obs per group:
                                                              min =          1
                                                              avg =        1.6
                                                              max =          7

Integration method: mvaghermite                 Integration pts.  =         12

                                                Wald chi2(3)      =       5.10
Log likelihood  = -186.36335                    Prob > chi2       =     0.1646

------------------------------------------------------------------------------
     infected | Odds Ratio   Std. Err.      z    P>|z|     [95% Conf. Interval]
-------------+----------------------------------------------------------------
close_contact |
          1  |   3.707632   2.323878     2.09   0.037     1.085372    12.66528
          2  |   3.828124   2.805632     1.83   0.067     .9102076    16.10021
          3  |   3.306103   2.006683     1.97   0.049     1.006163    10.86336
             |
        _cons |   4.806467   2.543582     2.97   0.003      1.70361    13.56069
-------------+----------------------------------------------------------------
     /lnsig2u |   1.435319   .5863963                      .2860032    2.584635
-------------+----------------------------------------------------------------
      sigma_u |    2.04963   .6009478                      1.153732    3.641214
          rho |   .5608152   .1444303                       .288056    .8011961
------------------------------------------------------------------------------
Note: Estimates are transformed only in the first equation.
Note: _cons estimates baseline odds (conditional on zero random effects).
LR test of rho=0: chibar2(01) = 12.45                  Prob >= chibar2 = 0.000

Refitting model intpoints() =  8
Refitting model intpoints() = 16

                         Quadrature check

             Fitted       Comparison     Comparison
           quadrature     quadrature     quadrature
           12 points      8 points       16 points
-----------------------------------------------------
Log        -186.36335     -186.40454     -186.37126
likelihood                -.04118703     -.00791285   Difference
                            .000221      .00004246   Relative difference
-----------------------------------------------------
infected:    1.3103933      1.354341      1.3121941
1.close_co~t               .04394772      .00180077   Difference
                           .03353781      .00137422   Relative difference
-----------------------------------------------------
infected:    1.3423749     1.3980193        1.34371
2.close_co~t               .05564439      .00133513   Difference
                            .0414522      .00099461   Relative difference
-----------------------------------------------------
infected:    1.1957702     1.2295811       1.196857
3.close_co~t               .03381092      .00108675   Difference
                           .02827543      .00090883   Relative difference
-----------------------------------------------------
infected:    1.5699622     1.6065224      1.5696796
   _cons                   .03656019     -.00028266   Difference
```

```
                           .02328731     -.00018004   Relative difference
-----------------------------------------------------
/:            1.4353188      1.5227711      1.4366071
 lnsig2u                     .08745226      .00128826   Difference
                             .0609288       .00089754   Relative difference
-----------------------------------------------------

 RECODE of |
diffpcrser |       INFECTED
         o |         0          1 |     Total
-----------+----------------------+----------
      <100 |         9         95 |       104
           |      8.65      91.35 |    100.00
-----------+----------------------+----------
   100-150 |        36        170 |       206
           |     17.48      82.52 |    100.00
-----------+----------------------+----------
      >150 |        25        119 |       144
           |     17.36      82.64 |    100.00
-----------+----------------------+----------
     Total |        70        384 |       454
           |     15.42      84.58 |    100.00

Random-effects logistic regression             Number of obs     =        454
Group variable: hh_id                          Number of groups  =        276

Random effects u_i ~ Gaussian                  Obs per group:
                                                             min =          1
                                                             avg =        1.6
                                                             max =          7

Integration method: mvaghermite                Integration pts.  =         12

                                               Wald chi2(2)      =       4.29
Log likelihood  = -186.63517                   Prob > chi2       =     0.1173

------------------------------------------------------------------------------
        infected | Odds Ratio   Std. Err.      z    P>|z|     [95% Conf. Interval]
-----------------+------------------------------------------------------------
diffpcrsero_cat3 |
        100-150  |    .276207   .1805619    -1.97   0.049     .0766986    .9946762
           >150  |   .2710302   .1847091    -1.92   0.055     .0712718    1.030666
                 |
           _cons |     36.349   28.04112     4.66   0.000     8.013837    164.8711
-----------------+------------------------------------------------------------
        /lnsig2u |   1.264507   .5691402                       .149013    2.380002
-----------------+------------------------------------------------------------
         sigma_u |   1.881847   .5355173                      1.077352    3.287084
             rho |   .5184066   .1420922                      .2607962    .7665897
------------------------------------------------------------------------------
Note: Estimates are transformed only in the first equation.
Note: _cons estimates baseline odds (conditional on zero random effects).
LR test of rho=0: chibar2(01) = 11.81                 Prob >= chibar2 = 0.000

Refitting model intpoints() =  8
Refitting model intpoints() = 16

                         Quadrature check

              Fitted       Comparison     Comparison
            quadrature     quadrature     quadrature
            12 points      8 points       16 points
-----------------------------------------------------
Log         -186.63517     -186.65771     -186.63755
likelihood                 -.0225399      -.00238026   Difference
                            .00012077      .00001275   Relative difference
-----------------------------------------------------
infected:   -1.2866048     -1.3056558     -1.2885305
2.diffpcrs~3               -.01905103     -.00192576   Difference
                            .01480721      .00149678   Relative difference
-----------------------------------------------------
infected:   -1.3055249     -1.3257099       -1.30756
3.diffpcrs~3               -.02018494     -.00203506   Difference
                            .01546117       .0015588   Relative difference
-----------------------------------------------------
infected:    3.5931667      3.6406295      3.5956352
   _cons                    .04746282      .00246852   Difference
```

```
                             .01320919        .000687   Relative difference
-----------------------------------------------------
/:            1.2645073      1.3067693      1.2657784
 lnsig2u                     .04226203      .00127112   Difference
                             .03342174      .00100523   Relative difference
-----------------------------------------------------

 RECODE of |       INFECTED
    dayshh |         0          1 |     Total
-----------+----------------------+----------
         0 |        14         42 |        56
           |     25.00      75.00 |    100.00
-----------+----------------------+----------
      1-3d |        17         66 |        83
           |     20.48      79.52 |    100.00
-----------+----------------------+----------
      4-7d |        22        115 |       137
           |     16.06      83.94 |    100.00
-----------+----------------------+----------
       >7d |        17        161 |       178
           |      9.55      90.45 |    100.00
-----------+----------------------+----------
     Total |        70        384 |       454
           |     15.42      84.58 |    100.00

Random-effects logistic regression              Number of obs     =        454
Group variable: hh_id                           Number of groups  =        276

Random effects u_i ~ Gaussian                   Obs per group:
                                                              min =          1
                                                              avg =        1.6
                                                              max =          7

Integration method: mvaghermite                 Integration pts.  =         12

                                                Wald chi2(3)      =       7.45
Log likelihood  = -184.41434                    Prob > chi2       =     0.0588

------------------------------------------------------------------------------
    infected | Odds Ratio   Std. Err.      z    P>|z|     [95% Conf. Interval]
-------------+----------------------------------------------------------------
  dayshh_cat |
       1-3d  |   1.308621   .8418139     0.42   0.676     .3708942    4.617188
       4-7d  |   2.475324   1.589671     1.41   0.158     .7030465    8.715252
        >7d  |   5.627842   3.942839     2.47   0.014     1.425589    22.21721
             |
       _cons |   4.992694   2.834152     2.83   0.005     1.641126    15.18896
-------------+----------------------------------------------------------------
    /lnsig2u |   1.283373   .5968239                      .1136198    2.453126
-------------+----------------------------------------------------------------
     sigma_u |   1.899682   .5668878                      1.058455    3.409492
         rho |   .5231149   .1488871                       .254031    .7794182
------------------------------------------------------------------------------
Note: Estimates are transformed only in the first equation.
Note: _cons estimates baseline odds (conditional on zero random effects).
LR test of rho=0: chibar2(01) = 11.22                  Prob >= chibar2 = 0.000

Refitting model intpoints() =  8
Refitting model intpoints() = 16

                         Quadrature check

             Fitted       Comparison     Comparison
             quadrature   quadrature     quadrature
             12 points    8 points       16 points
-----------------------------------------------------
Log          -184.41434   -184.43371     -184.41696
likelihood                -.01936883      -.0026269   Difference
                           .00010503      .00001424   Relative difference
-----------------------------------------------------
infected:     .26897372    .26949963      .26894139
1.dayshh_cat               .00052591     -.00003233   Difference
                           .00195523     -.00012021   Relative difference
-----------------------------------------------------
infected:     .90637119    .92567195      .90733675
2.dayshh_cat               .01930076      .00096556   Difference
                           .02129454       .0010653   Relative difference
```

```
-----------------------------------------------------
infected:     1.7277261      1.7565796       1.730153
3.dayshh_cat                  .02885345      .00242684   Difference
                              .01670024      .00140465   Relative difference
-----------------------------------------------------
infected:     1.6079757      1.6282628       1.608615
   _cons                      .02028706      .00063934   Difference
                              .01261652      .00039761   Relative difference
-----------------------------------------------------
/:            1.2833731      1.3332178       1.285901
 lnsig2u                      .04984464      .00252788   Difference
                              .03883877      .00196971   Relative difference
-----------------------------------------------------

.
```

10. Summary of VOC and infection - unadjusted

```
.
. tab vocnew infected, row

           |       INFECTED
    vocnew |         0          1 |     Total
-----------+----------------------+----------
   Non-VOC |        25         77 |       102
           |     24.51      75.49 |    100.00
-----------+----------------------+----------
       VOC |        45        307 |       352
           |     12.78      87.22 |    100.00
-----------+----------------------+----------
     Total |        70        384 |       454
           |     15.42      84.58 |    100.00

. xtlogit infected i.vocnew, re or nolog/* Random effects logistic. [3.54] NB: rho is large
(0.33) so numerical approx should be checked*/

Random-effects logistic regression              Number of obs     =        454
Group variable: hh_id                           Number of groups  =        276

Random effects u_i ~ Gaussian                   Obs per group:
                                                              min =          1
                                                              avg =        1.6
                                                              max =          7

Integration method: mvaghermite                 Integration pts.  =         12

                                                Wald chi2(1)      =       5.33
Log likelihood  = -186.37494                    Prob > chi2       =     0.0210

------------------------------------------------------------------------------
    infected | Odds Ratio   Std. Err.      z    P>|z|     [95% Conf. Interval]
-------------+----------------------------------------------------------------
      vocnew |
        VOC  |   3.134187   1.551193     2.31   0.021     1.188071    8.268132
       _cons |   5.060994   2.296476     3.57   0.000     2.079672     12.3162
-------------+----------------------------------------------------------------
    /lnsig2u |    1.17917   .5982633                      .0065957    2.351745
-------------+----------------------------------------------------------------
     sigma_u |    1.80324   .5394062                      1.003303    3.240969
         rho |   .4970807   .1495607                      .2342886    .7614957
------------------------------------------------------------------------------
Note: Estimates are transformed only in the first equation.
Note: _cons estimates baseline odds (conditional on zero random effects).
LR test of rho=0: chibar2(01) = 9.97                   Prob >= chibar2 = 0.001

. quadchk, nooutput

Refitting model intpoints() =  8
Refitting model intpoints() = 16

                         Quadrature check

             Fitted         Comparison     Comparison
             quadrature     quadrature     quadrature
             12 points      8 points       16 points
```

```
----------------------------------------------------
Log            -186.37494     -186.39031     -186.37744
likelihood                    -.01537189     -.00249734   Difference
                               .00008248       .0000134   Relative difference
----------------------------------------------------
infected:       1.1423699      1.1598928      1.1428786
1.vocnew                       .01752297      .00050875   Difference
                               .01533914      .00044535   Relative difference
----------------------------------------------------
infected:        1.621563      1.6375047      1.6213921
   _cons                       .01594172     -.00017092   Difference
                               .00983108      -.0001054   Relative difference
----------------------------------------------------
/:              1.1791702      1.2212503      1.1792722
 lnsig2u                       .04208005      .00010197   Difference
                               .03568615      .00008648   Relative difference
----------------------------------------------------
```

11. Summary of VOC and infection – adjusted (with continuous covariates + age assessed using fractional polynomals); plsu adjustment for close contact (as a factor)

```
.
. * fractional polynomials for continuous terms for agenewm, diffpcrsero and dayshh
. mfp: xtlogit infected vocnew sexnew agenew diffpcrsero dayshh  imd_rank close_contacta
close_contactb close_contactc, re or  nolog

Deviance for model with all terms untransformed =   358.230, 454 observations

Variable     Model (vs.)   Deviance  Dev diff.   P      Powers   (vs.)
----------------------------------------------------------------------
agenew       lin.   FP2     358.230     4.078  0.253    1         .5 1
             Final          358.230                     1

[close_contactc included with 1 df in model]

[close_contactb included with 1 df in model]

[close_contacta included with 1 df in model]

[vocnew included with 1 df in model]

diffpcrsero  lin.   FP2     358.230     2.482  0.479    1         -2 -2
             Final          358.230                     1

[sexnew included with 1 df in model]

imd_rank     lin.   FP2     358.230     0.368  0.947    1         1 1
             Final          358.230                     1

dayshh       lin.   FP2     358.230     5.165  0.160    1         1 2
             Final          358.230                     1


Fractional polynomial fitting algorithm converged after 1 cycle.

Transformations of covariates:

-> gen double Iagen__1 = agenew-41.10352423 if e(sample)
-> gen double Idiff__1 = diffpcrsero-130.7400881 if e(sample)
-> gen double Idays__1 = dayshh-8.372246696 if e(sample)
-> gen double Iimd___1 = imd_rank-11221.59251 if e(sample)

Final multivariable fractional polynomial model for infected
--------------------------------------------------------------------
    Variable |    -----Initial-----          -----Final-----
             |   df     Select   Alpha    Status    df    Powers
-------------+------------------------------------------------------
      vocnew |    1     1.0000   0.0500     in       1     1
      sexnew |    1     1.0000   0.0500     in       1     1
      agenew |    4     1.0000   0.0500     in       1     1
 diffpcrsero |    4     1.0000   0.0500     in       1     1
      dayshh |    4     1.0000   0.0500     in       1     1
```

```
    imd_rank |     4      1.0000    0.0500     in       1      1
close_con... |     1      1.0000    0.0500     in       1      1
close_con... |     1      1.0000    0.0500     in       1      1
close_con... |     1      1.0000    0.0500     in       1      1
--------------------------------------------------------------------

Random-effects logistic regression               Number of obs     =        454
Group variable: hh_id                            Number of groups  =        276

Random effects u_i ~ Gaussian                    Obs per group:
                                                              min =          1
                                                              avg =        1.6
                                                              max =          7

Integration method: mvaghermite                  Integration pts.  =         12

                                                 Wald chi2(9)      =      12.79
Log likelihood  = -179.11476                     Prob > chi2       =     0.1723

--------------------------------------------------------------------------------
      infected | Odds Ratio   Std. Err.      z    P>|z|     [95% Conf. Interval]
---------------+----------------------------------------------------------------
        vocnew |   2.342272   1.286055     1.55   0.121     .7984979    6.870696
        sexnew |   .7827221   .3016591    -0.64   0.525     .3677533    1.665937
      Iagen__1 |   .9782973   .0096063    -2.23   0.025     .9596493    .9973076
      Idiff__1 |   .9916954    .006634    -1.25   0.213     .9787779    1.004783
      Idays__1 |     1.0134   .0286187     0.47   0.637     .9588321    1.071073
      Iimd___1 |   .9999853   .0000284    -0.52   0.605     .9999297    1.000041
 close_contacta |   3.208471   2.036449     1.84   0.066     .9247665    11.13177
 close_contactb |   4.814071   3.761967     2.01   0.044     1.040732    22.26824
 close_contactc |   3.959331   2.639507     2.06   0.039     1.071924    14.62445
         _cons |   2.809021    1.84322     1.57   0.115     .7762733    10.16472
---------------+----------------------------------------------------------------
      /lnsig2u |   1.280453   .6386182                      .0287844    2.532122
---------------+----------------------------------------------------------------
       sigma_u |   1.896911   .6057008                      1.014496    3.546853
           rho |   .5223864   .1593345                      .2382926    .7926994
--------------------------------------------------------------------------------
Note: Estimates are transformed only in the first equation.
Note: _cons estimates baseline odds (conditional on zero random effects).
LR test of rho=0: chibar2(01) = 9.45                  Prob >= chibar2 = 0.001
Deviance:  358.230.
```

12. Summary of VOC and infection – adjusted (with continuous covariates + age assessed using fractional polynomals); plus adjustment for close contact (as a quantitative variable)

```
. mfp: xtlogit infected vocnew sexnew agenew diffpcrsero dayshh imd_rank close_contact, re or
nolog

Deviance for model with all terms untransformed =   361.053, 454 observations

Variable      Model  (vs.)    Deviance  Dev diff.    P      Powers    (vs.)
----------------------------------------------------------------------------
agenew        lin.   FP2       361.053     4.820   0.185    1          .5 1
              Final            361.053                      1

close_con...  lin.   FP1       361.053     2.619   0.106    1          -2
              Final            361.053                      1

[vocnew included with 1 df in model]

diffpcrsero   lin.   FP2       361.053     2.812   0.422    1          -2 -2
              Final            361.053                      1

imd_rank      lin.   FP2       361.053     0.401   0.940    1          1 1
              Final            361.053                      1

dayshh        lin.   FP2       361.053     6.643   0.084    1          1 2
              Final            361.053                      1
```

```
[sexnew included with 1 df in model]


Fractional polynomial fitting algorithm converged after 1 cycle.

Transformations of covariates:

-> gen double Iagen__1 = agenew-41.10352423 if e(sample)
-> gen double Idiff__1 = diffpcrsero-130.7400881 if e(sample)
-> gen double Idays__1 = dayshh-8.372246696 if e(sample)
-> gen double Iimd___1 = imd_rank-11221.59251 if e(sample)
-> gen double Iclos__1 = close_contact-1.698237885 if e(sample)

Final multivariable fractional polynomial model for infected
--------------------------------------------------------------------
    Variable |    -----Initial-----          -----Final-----
             |   df     Select   Alpha    Status    df    Powers
-------------+------------------------------------------------------
      vocnew |    1     1.0000   0.0500     in      1     1
      sexnew |    1     1.0000   0.0500     in      1     1
      agenew |    4     1.0000   0.0500     in      1     1
 diffpcrsero |    4     1.0000   0.0500     in      1     1
      dayshh |    4     1.0000   0.0500     in      1     1
    imd_rank |    4     1.0000   0.0500     in      1     1
close_con... |    2     1.0000   0.0500     in      1     1
--------------------------------------------------------------------

Random-effects logistic regression              Number of obs     =        454
Group variable: hh_id                           Number of groups  =        276

Random effects u_i ~ Gaussian                   Obs per group:
                                                              min =          1
                                                              avg =        1.6
                                                              max =          7

Integration method: mvaghermite                 Integration pts.  =         12

                                                Wald chi2(7)      =      12.81
Log likelihood  = -180.52669                    Prob > chi2       =     0.0769

------------------------------------------------------------------------------
    infected | Odds Ratio   Std. Err.      z    P>|z|     [95% Conf. Interval]
-------------+----------------------------------------------------------------
      vocnew |   2.114488   1.069077     1.48   0.139     .7849484    5.695993
      sexnew |   .7891921   .2892698    -0.65   0.518     .3847548    1.618756
    Iagen__1 |   .9794929   .0088218    -2.30   0.021     .9623542    .9969369
    Idiff__1 |    .991273   .0062188    -1.40   0.162      .979159    1.003537
    Idays__1 |   1.017929   .0272873     0.66   0.507     .9658271     1.07284
    Iimd___1 |   .9999826    .000026    -0.67   0.504     .9999317    1.000034
    Iclos__1 |   1.412299   .2738079     1.78   0.075     .9658307    2.065154
       _cons |   8.211732   4.589491     3.77   0.000     2.746016    24.55651
-------------+----------------------------------------------------------------
    /lnsig2u |   1.069111   .6310277                     -.1676807    2.305903
-------------+----------------------------------------------------------------
     sigma_u |   1.706689   .5384842                      .9195781    3.167527
         rho |   .4696034   .1571739                      .2044796    .7530703
------------------------------------------------------------------------------
Note: Estimates are transformed only in the first equation.
Note: _cons estimates baseline odds (conditional on zero random effects).
LR test of rho=0: chibar2(01) = 8.20                   Prob >= chibar2 = 0.002
Deviance:  361.053.

.
```

13. Summary of VOC and infection – adjusted (with continuous covariates + age assessed using fractional polynomals); no adjustment for close contact

```
. mfp: xtlogit infected vocnew sexnew agenew diffpcrsero dayshh  imd_rank , re or  nolog

Deviance for model with all terms untransformed =   364.540, 454 observations

Variable     Model (vs.)    Deviance  Dev diff.   P      Powers   (vs.)
```

```
-------------------------------------------------------------------
agenew       lin.   FP2     364.540     3.859  0.277    1         .5 .5
             Final          364.540                     1

[vocnew included with 1 df in model]

diffpcrsero  lin.   FP2     364.540     3.082  0.379    1         -2 -2
             Final          364.540                     1

dayshh       lin.   FP2     364.540     8.447  0.038+   1         1 2
             FP1            360.471     4.378  0.112    -.5
             Final          360.471                     -.5

imd_rank     lin.   FP2     360.471     0.191  0.979    1         1 2
             Final          360.471                     1

[sexnew included with 1 df in model]

-------------------------------------------------------------------
End of Cycle 1: deviance =     360.471
-------------------------------------------------------------------

agenew       lin.   FP2     360.471     4.392  0.222    1         .5 1
             Final          360.471                     1

[vocnew included with 1 df in model]

diffpcrsero  lin.   FP2     360.471     2.890  0.409    1         -2 -2
             Final          360.471                     1

dayshh       lin.   FP2     364.540     8.447  0.038+   1         1 2
             FP1            360.471     4.378  0.112    -.5
             Final          360.471                     -.5

imd_rank     lin.   FP2     360.471     0.191  0.979    1         1 2
             Final          360.471                     1

[sexnew included with 1 df in model]


Fractional polynomial fitting algorithm converged after 2 cycles.

Transformations of covariates:

-> gen double Iagen__1 = agenew-41.10352423 if e(sample)
-> gen double Idiff__1 = diffpcrsero-130.7400881 if e(sample)
-> gen double Idays__1 = X^-.5-1.032947251 if e(sample)
   (where: X = (dayshh+1)/10)
-> gen double Iimd___1 = imd_rank-11221.59251 if e(sample)

Final multivariable fractional polynomial model for infected
--------------------------------------------------------------------
    Variable |    -----Initial-----          -----Final-----
             |   df     Select   Alpha    Status    df    Powers
-------------+------------------------------------------------------
      vocnew |    1     1.0000   0.0500     in      1     1
      sexnew |    1     1.0000   0.0500     in      1     1
      agenew |    4     1.0000   0.0500     in      1     1
 diffpcrsero |    4     1.0000   0.0500     in      1     1
      dayshh |    4     1.0000   0.0500     in      2     -.5
    imd_rank |    4     1.0000   0.0500     in      1     1
--------------------------------------------------------------------

Random-effects logistic regression              Number of obs     =        454
Group variable: hh_id                           Number of groups  =        276

Random effects u_i ~ Gaussian                   Obs per group:
                                                              min =          1
                                                              avg =        1.6
                                                              max =          7

Integration method: mvaghermite                 Integration pts.  =         12

                                                Wald chi2(6)      =      13.17
Log likelihood  = -180.23557                    Prob > chi2       =     0.0404

------------------------------------------------------------------------------
```

```
    infected | Odds Ratio   Std. Err.      z    P>|z|     [95% Conf. Interval]
-------------+----------------------------------------------------------------
      vocnew |   1.982537   1.002312     1.35   0.176     .7360017    5.340275
      sexnew |    .855008   .3079181    -0.43   0.664     .4221073    1.731879
    Iagen__1 |    .983049   .0082945    -2.03   0.043     .9669258    .9994411
    Idiff__1 |   .9921711   .0060788    -1.28   0.200     .9803281    1.004157
    Idays__1 |   .5641195   .1477865    -2.19   0.029     .3375792    .9426849
    Iimd___1 |   .9999812   .0000257    -0.73   0.466     .9999309    1.000032
       _cons |   10.15828   6.011574     3.92   0.000     3.184837    32.40061
-------------+----------------------------------------------------------------
    /lnsig2u |   1.042304    .640488                     -.2130291    2.297638
-------------+----------------------------------------------------------------
     sigma_u |   1.683967   .5392803                       .898962    3.154465
         rho |   .4629324    .159242                      .1972017    .7515303
------------------------------------------------------------------------------
Note: Estimates are transformed only in the first equation.
Note: _cons estimates baseline odds (conditional on zero random effects).
LR test of rho=0: chibar2(01) = 7.86                   Prob >= chibar2 = 0.003
Deviance:  360.471.
```

14. Summary of VOC and infection

	% infection (n/N)	Unadjusted OR (95% CI)	P-value	Adjusted OR*(95% CI)	P-value
Non-VOC	75% (77/102)				
VOC	87% (307/352)	3.1 (1.2-8.3)	0.02	2.1 (0.8-5.5)	0.14

* adjusted for sex, age (years; linear), time since pcr_date date to date of HH serology (days; linear), infectious index and HHC in contact (days; linear), IMD (rank; linear), close contacts (4 levels assuming a linear trend)

Table 3. Co-variate impact on seropositivity of household contacts in a multivariate logistic regression model, results displayed for both unadjusted model and adjusted for household clustering

			**Model 1 NC positive as outcome n=352 Adjusted Odds Ratio**			**Model 2 All NC positive & Unvaccinated Spike positive as outcome n=384 Adjusted Odds Ratio**		
			**NC positive**	**aOR(95%CI)**	**p-value**	**All infected**	**aOR(95%CI)**	**p-value**
**Index**	**NonVOC SARS-CoV-2**		63/102	*baseline		77/102		
	**VOC SARS-CoV-2**		289/352	2.26 (1.03-4.90)	0.04	307/352		
	**Days at home whilst symptomatic Median (IQR)**		7(3-12)	1.02 (0.98-1.07)	0.3	7(3-12)		
	**IMD quartile**	**Lowest (1-2.5)**	81/107	*baseline		89/107		

		**Second (>2.5-5)**	187/238	1.44 (0.70-2.96)	0.3	206/238		
		**Third (>5-7.5)**	33/37	1.58 (0.49-5.07)	0.4	34/37		
		**Highest (>7.5-10)**	51/72	1.27 (0.48-3.34)	0.6	55/72		
	**Time since index PCR diagnosis**		130 (101-159)	0.99 (0.98-0.99)	0.04	130 (101-159)		
**Contact**	**sex**	**female**	202/261	baseline		219/261		
		**male**	150/193	1.13 (0.64-1.98)	0.7	165/193		
**Intensity of Contact****	**No close contact within home**		44/68	*baseline		51/68		
	**Shared bathroom**		118/157	1.94 (0.80-4.69)	0.14	136/157		
	**Shared bedroom**		61/75	2.98 (1.04-8.58)	0.04	64/75		
	**Help with personal care**		129/154	3.38 (1.31-8.75)	0.01	133/154		

Table. 1
Baseline characteristics stratified by Indices VOC and non-VOC SARS-CoV-2 variant status for whom we have serum Total N = 454

	Non-VOC index case household	Alpha index case household	p-value*
Number of index - n	50	188	
Number of contacts - n	102	352	
**Index characteristics**			
index case female - n (%)	26 (52)	111 (59)	0.3

Index case age – median (IQR)		56 (45-72)	57 (47-69)	0.9
ethnicity	white (%)	27 (54)	96 (51)	0.3
	Asian (%)	2 (4)	14 (8)	
	Black (%)	7 (14)	34 (18)	
	Middle Eastern (%)	5 (10)	15 (6)	
	SE Asian (%)	7 (14)	13 (7)	
	other	2 (4)	8 (4.3)	
Hospital site	Hospital 1	49 (48)	173 (49)	0.8
	Hospital 2	53 (52)	179 (51)	
household size[1] – median (IQR)		3 (2-4)	3 (2-4)	>0.99
index case respiratory symptoms – n (%)		35 (70)	146 (78)	0.2
symptom duration in days – median(IQR)		7 (3-10)	7 (5-114)	0.2
index case hospitalisation – n (%)		29 (58)	124 (66)	0.3
index case ICU admission – n (%)		5 (17)	35 (29)	0.3
index case mortality – n(%)		1 (2)	2 (1)	0.6
IMD decile (IQR)		3 (2-6)	3 (2-5)	0.7
Time since index PCR diagnosis -median (IQR)		146 (125-181)	119 (93-157)	<0.001

Contact characteristics				
contact female – n (%)		57 (56)	204 (58)	0.7
contact age – median (IQR)		44 (22-59)	42 (24-60)	0.9
days of exposure to index – median (IQR)		4 (1-10)	7 (3-14)	<0.001
proximity to index – n (%)	no close contact	19(19)	49(14)	0.06
	assisted in personal care	24 (24)	130 (37)	
	shared bedroom	16 (16)	59 (17)	
	shared bathroom	43 (42)	114 (32)	
other covid exposure (not index) – n (%)		26 (26)	107 (30)	0.3
previous COVID-19 diagnosis – n (%)		48 (47)	235 (67)	<0.001
Contact symptoms - n (%)		40 (39)	183 (52)	0.045
Long COVID - n (%)		4 (8)	8 (4)	0.1
vaccination status at time of serum sampling	unvaccinated	49 (48)	169 (48)	0.4
	single vaccination	24 (24)	102 (29)	
	double vaccination	29 (28)	81 (23)	

Supplementary 1. Baseline Characteristics in participants with and without serum samples

		Participants without serum samples	Participants with serum samples	p-value*
Number of index - n		200	281	
Number of contacts - n		385	454	
Alpha index		162	188	
non-VOC index		38	50	
**Index characteristics**				
index case female - n (%)		220 (57.1%)	255 (56.2%)	0.3
Index case age – median (IQR)		51 (40- 64)	57 (47-70)	0.9
ethnicity	white (%)	27 (54)	96 (51)	0.3
	Asian (%)	2 (4)	14 (8)	
	Black (%)	7 (14)	34 (18)	
	Middle Eastern (%)	5 (10)	15 (6)	
	SE Asian (%)	7 (14)	13 (7)	
	other	2 (4)	8 (4.3)	
Hospital site	Hospital 1	49 (48)	173 (49)	0.8
	Hospital 2	53 (52)	179 (51)	
household size[1] – median (IQR)		3 (2-4)	3 (2-4)	>0.99

index case respiratory symptoms – n (%)		35 (70)	146 (78)	0.2
symptom duration in days – median(IQR)		7 (3-10)	7 (4-12)	0.4
index case hospitalisation – n (%)		29 (58)	124 (66)	0.3
index case ICU admission – n (%)		5 (17)	35 (29)	0.3
index case mortality – n(%)		1 (2)	2 (1)	0.6
IMD decile (IQR)		3 (2-6)	3 (2-5)	0.7
Time since index PCR diagnosis - median (IQR)		139(111-164)	130 (101-158)	0.003
**Contact characteristics**				
contact female – n (%)		172 (45%)	204 (58)	0.7
contact age – median (IQR)		25 (12-45)	42 (24-60)	<0.001
days of exposure to index – median (IQR)		5 (2-10)	7 (3-14)	0.004
proximity to index – n (%)	no close contact	86(22)	49(14)	<0.001
	assisted in personal care	167 (43)	130 (37)	
	shared bedroom	37 (10)	59 (17)	
	shared bathroom	97 (25)	114 (32)	
other covid exposure (not index) – n (%)		26 (26)	107 (30)	0.3

previous COVID-19 diagnosis – n (%)		48 (47)	235 (67)	<0.001
Contact symptoms - n (%)		40 (39)	183 (52)	0.045
Long COVID - n (%)		4 (8)	8 (4)	0.1
vaccination status at time of serum sampling	unvaccinated	260(68)	169 (48)	<0.001
	single vaccination	68(18)	102 (29)	
	double vaccination	57(15)	81 (23)	

Supplementary Figure 1
Correlation of antibody titres between
(i) Log normalised alpha VOC vrs non-VOC SARS-CoV-2 spike protein
(ii) Log normalised alpha VOC vrs non-VOC SARS-CoV-2 RBD

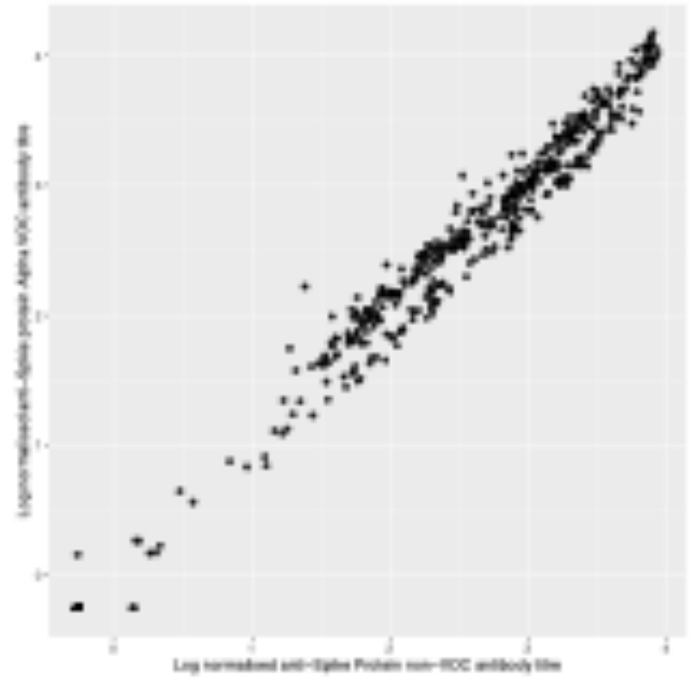

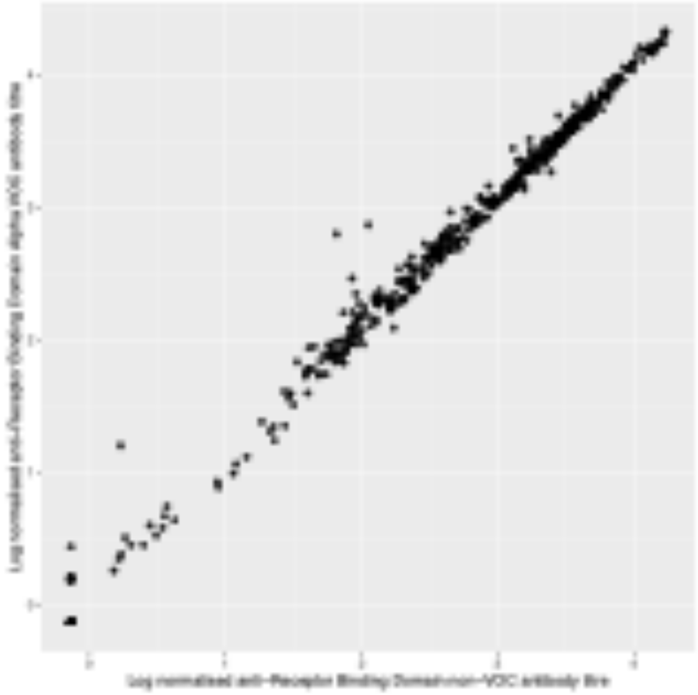

Supplement: S1 File — (PDF) [file pone.0284372.s002.pdf]
