# Supplementary material for: Comparison of new and emerging SARS-CoV-2 variant transmissibility through active contact testing. A comparative cross-sectional household seroprevalence study
Source: PLoS One. 2023 Apr 24;18(4):e0284372. doi: 10.1371/journal.pone.0284372 (PMC10124829; doi:10.1371/journal.pone.0284372)
Supplement: Attachment — Submitted filename: PONE-D-22-28360-review.pdf [file pone.0284372.s004.pdf]

Review PONE-D-22-28368

November 11, 2022

This is a very nice study that demonstrates convincingly the higher transmissibility of the alpha variant of SARS-CoV-2 as compared to the non-VOC strain. The data supporting this comes from a serological survey which is extremely useful as it elimintes bias that comes from only counting symptomatic individuals as is the usual practice. My main concern with this article is that the methodology is barely explained at all nor is any software code available. Though the general technique is standard, a more thorough explanation is required because there can be devils in those details: in this case the obvious one is how censorship is handled. It would also be useful to report SITP alongside SAR.

Major comments

- There is neither an explanation of the statistical methodology (e.g. in math) nor any code made available that I could find from which to understand precisely how the analysis was carried out. I would expect both to be provided. It is essential to provide this information because without it there is no way to understand what statements like "OR are adjusted for household level clustering" mean and likewise how censoring (see next point) was handled. It isn't enough to say that the code is only useful with the data and the data is only available upon request.
- Of the households for which an index case took part, it seems that not all contacts also took part and of those who did, not all had serology available. In other words, there is clearly censoring in the data. Indeed 454 contacts fully participated (line 169) and 386 declined serology but filled in a survey (line 174). It is unclear from the methodology how

this censoring was handled in the analysis. We should be able to get upper (assume all of those who declined became infected) and lower (assume none of them became infected) bounds on the estimates of SAR from this.

- Though SAR is a standard measure in the field, SITP (susceptible-infectious transmission probability) is more useful especially when comparing across studies. Ideally this could be computed and reported for each household size. This is only a suggestion but I think it would make this study stronger.

Minor comments

- Definition of duration of exposure is somewhat problematic because it doesn't admit the possibility of presymptomatic transmission. However, I think it is likely that trying to fix this could significantly complicate the analysis without substantially affecting the overall result. Suggest to simply mention this.

- Representativeness. Study population 56% female, median age 42 years, etc. One would expect to report these figures alongside the corresponding ones for the population in the study geography as a whole to help judge the possibility of bias. Such bias, if present, could be attributable to sampling or to differential circulation of the virus in different subpopulations. It is unlikely to be possible to tell from the data collected but it is still important to know. These figures for the background demographics should be readily available so reporting them shouldn't be a significant burden.
